# A Genome-Wide Landscape of Retrocopies in Primate Genomes

**DOI:** 10.1093/gbe/evv142

**Published:** 2015-07-29

**Authors:** Fábio C.P. Navarro, Pedro A.F. Galante

**Affiliations:** ^1^Centro de Oncologia Molecular, Hospital Sírio-Libanês, São Paulo, Brazil; ^2^Dep. de Bioquímica, Universidade de São Paulo, Brazil

**Keywords:** retrocopy, primate genomes, gene duplication, retrogene

## Abstract

Gene duplication is a key factor contributing to phenotype diversity across and within species. Although the availability of complete genomes has led to the extensive study of genomic duplications, the dynamics and variability of gene duplications mediated by retrotransposition are not well understood. Here, we predict mRNA retrotransposition and use comparative genomics to investigate their origin and variability across primates. Analyzing seven anthropoid primate genomes, we found a similar number of mRNA retrotranspositions (∼7,500 retrocopies) in Catarrhini (Old Word Monkeys, including humans), but a surprising large number of retrocopies (∼10,000) in Platyrrhini (New World Monkeys), which may be a by-product of higher long interspersed nuclear element 1 activity in these genomes. By inferring retrocopy orthology, we dated most of the primate retrocopy origins, and estimated a decrease in the fixation rate in recent primate history, implying a smaller number of species-specific retrocopies. Moreover, using RNA-Seq data, we identified approximately 3,600 expressed retrocopies. As expected, most of these retrocopies are located near or within known genes, present tissue-specific and even species-specific expression patterns, and no expression correlation to their parental genes. Taken together, our results provide further evidence that mRNA retrotransposition is an active mechanism in primate evolution and suggest that retrocopies may not only introduce great genetic variability between lineages but also create a large reservoir of potentially functional new genomic loci in primate genomes.

## Introduction

Gene duplication is a major contributor to the origin of adaptive evolutionary novelties (Ohno 1970; Long et al. 2003). Small-scale duplications can be created by chromosome segmental duplications, a DNA-mediated mechanism (reviewed in [Prince and Pickett 2002; Marques-Bonet, Girirajan, et al. 2009]), or through reverse transcription of mature RNA intermediates, a mechanism known as retrotransposition or retroduplication of mRNAs (Esnault et al. 2000). Although the former mechanism has been extensively studied (Zhang 2003; Sharp et al. 2005; Conrad and Antonarakis 2007), the impact and extent of retroduplication of the mRNAs still deserves a deep and systematic investigation in many species (Kaessmann et al. 2009).

In eutheria, mRNA retroduplication is carried out by two long interspersed nuclear element 1 (LINE1 [L1]) proteins: one protein that exhibits reverse-transcriptase (Mathias et al. 1991) and endonuclease (Feng et al. 1996) activities and an RNA-binding protein (Hohjoh and Singer 1997). Together, the two proteins hijack RNAs in the cytoplasm, synthesize (retro)copies, and integrate the resultant transcripts into the nuclear genome (Esnault et al. 2000). Therefore, mRNA retrocopies usually contain only exonic sequences, lacking introns and the upstream regulatory regions of their parental genes, and have been classified by some authors as dead on arrival “processed pseudogenes” (McClintock 1953; Vanin 1985; Zhang et al. 2004). However, despite the absence of regulatory regions, since the late 1980s (McCarrey and Thomas 1987; Marques et al. 2005a; Mandal and Kazazian 2008), there has been growing evidence that many retrocopies are in fact functional (usually called retrogenes) and may also have noncoding transcripts (Trembley et al. 2005; Galante et al. 2007; Baertsch et al. 2008; Tam et al. 2008; Hung et al. 2010; Poliseno et al. 2010; Fairbanks et al. 2012). Therefore, the term mRNA retrocopy (or simply retrocopy) is a general term that comprises processed pseudogenes and retrogenes.

Currently, the prediction of retrocopies in entire sequenced genomes relies on the identifications of intronless duplications of multiexonic genes, known as parental genes. However, due to differences in retrocopy screening strategies (Baertsch et al. 2008), there is no consensus on the number of retrocopies, even for the human genome. Methods based on mRNA sequence alignments and accurate annotations have identified 7,000–13,000 retrocopies (Sakai et al. 2007; Baertsch et al. 2008; Pei et al. 2012). However, methods based on protein sequence alignments have reported 3,000–6,000 retrocopies (Marques et al. 2005b; Vinckenbosch et al. 2006).

A remarkable feature of primate genomes is the proportion of retroposed insertions involving LINEs, short interspersed nuclear elements (SINEs) and other mobile elements, which account for up to approximately 45% of the genomes of humans (Lander et al. 2001; Venter et al. 2001), chimpanzees (Chimpanzee Sequencing and Analysis Consortium 2005), and gorillas (Scally et al. 2012). Because mRNA retrocopies are a subclass of retroposed copies and a potential source of novel functional transcripts, it is reasonable to hypothesize that they may play key roles in the primate genome evolution. Although some studies have explored retrocopies in primates, many of their features remain to be elucidated (Kaessmann et al. 2009).

Here, we performed a systematic analysis of mRNA retrocopies in seven fully sequenced primates and two murine rodent genomes (our “outgroup”). Specifically, we catalogued their entire retrocopy repertoires and explored the origin and orthology of the retrocopies and the potentially expressed retrocopies. We found that mRNA retrotranspositions are more frequent in New World Monkey (NWMs) than in Old World Monkeys (OWMs) genomes, and we confirmed that most primate retrocopies originated from their own lineage, with approximately 50% of retrocopies shared among all primates. We also identified a set of expressed and potentially functional retrocopies that exhibited tissue- and species-specific expressions.

## Materials and Methods

### Data Sources

The primate genome and transcriptome data sets were downloaded from the UCSC genome browser (Karolchik et al. 2014) and the RefSeq database (Pruitt et al. 2014): version 49 (human [GRCh37/hg19], mouse [mm9] and rat [rn4]); version 50 (chimpanzee [GCA_000001515.3/panTro3]); version 51 (orangutan [P_pygmaeus_2.0.2/ponAbe2], marmoset [GCA_000004665.1/calJac3], rhesus [GCA_000230795.1/rheMac2]); version 61 (squirrel monkey [SaiBol1.0]). Only the gorilla transcripts were downloaded from ENSEMBL (Flicek et al. 2014) (version 66) and the gorilla genome was also download from UCSC genome browser (GCA_000151905.1/gorGor3). The genomic coordinates for: 1) transcription start sites (TSS; GENCODE v12); 2) repetitive elements, polyadenylation (polyA) sites, and centromeric-telomeric regions were also obtained from the UCSC Genome Browser and used in the retrocopy genomic analysis. Finally, to investigate the expressed retrocopies, we used publicly available RNA-Seq data [GEO: GSE30352] generated by Brawand et al. (2011) for six tissues (brain, cerebellum, heart, liver, kidney, and testis) of five primates (human, chimpanzee, gorilla, orangutan, and rhesus).

### Identifying Retrocopies of Protein-Coding Genes

Because retrocopies are processed copies of multiexonic genes, our pipeline relied on the identification of genomic intronless alignments from mature transcripts (mRNAs). First, all known coding gene transcripts mRNAs were aligned to their respective reference genome using BLAT (parameters: -mask=lower; -tileSize=12; -minIdentity=75; -minScore=100). Next, we selected alignments with an identity greater than 75% such that either more than 50% of the parental transcript or at least 120 nt aligned. Alignments containing gaps larger than 15 kb (putatively large introns) were excluded from further analyses, this last filter removed most of the introns and allowed for some repetitive element insertions (generally <10 kb in length) inside the putative retrocopy loci. Next, we selected the retrocopies by screening for parental exons in each putative retroduplication event and selecting only those candidates with at least two parental exons adjacently aligned (>50 nt each). A random set of 200 human retrocopies and their parental genes was analyzed manually, less than 3% were estimated as potential false positives. For example, olfactory receptors and other problematic transcripts were manually removed from the final data set. Retrocopies of single exon genes were not detectable using our methodology. More details regarding this pipeline and primate retrocopies can be found in Navarro and Galante (2013).

### Characterization of the L1 Family

To better understand the large number of retrocopies present in the marmoset and squirrel monkey genomes, we compared the compositions of L1 subfamilies and the content and length of the L1 elements from all of the primate genomes using RepeatMasker data (Smit AFA, Hubley R, and Green P. RepeatMasker Open-3.0., http://www.repeatmasker.org). Due to the high content of L1 elements, only subfamilies with more than 10,000 members in the seven primates were analyzed. To analyze L1PA7 and L1P3 expansion in the NWM genomes, we initially selected L1PA7 elements with intact open reading frame 2 (ORF2) regions in all of the primate genomes, and we conducted a multiple alignment of DNA sequences of their ORF2 regions using CLUSTALW2 (parameters: -type=dna -quicktree). Finally, we plotted the phylogenetic tree, coloring each leaf according to species color, using iTOL (Letunic and Bork 2011).

### Inferring Shared and Species-Specific Retrotransposition Events

To infer the retrocopy origins among primates, instead of using the number of nonsynonymous mutations (Ohshima et al. 2003), which represents indirect evidence, we developed a strategy to select orthologous retroduplications events based on their syntenic genomic position, allowing us to fully assess the sequenced genomes and to define flanking sequences of retroduplication events (Scally et al. 2012). We defined a flanking region as the 3,000 nt adjacent to each retrocopy and composed of blocks with at least 150 nt of nonrepetitive sequences. To ensure that retrocopy segments were not included within the flanking regions, we started extracting flanking sequences 5,000 nt up- and downstream of each retrocopy event. Next, we performed a pairwise comparison between all species, aligning retrocopies, and their flanking regions using BLAT (parameters: -mask=lower; -tileSize=12; -minScore=50; -minIdentity=0). Events sharing the flanking regions and containing the same parental retrocopies as the query genomes were classified as orthologous. In contrast, after the pairwise comparisons, the unshared retrocopies were classified as species-specific retrotransposition events. This strategy has also been previously applied to identify ortholog events in our retrocopy database, RCPedia (Navarro and Galante 2013). It was used for all primates (human, chimpanzee, gorilla, orangutan, rhesus, marmoset, and squirrel monkey) and rodent (mouse and rat) genomes to identify shared and species-specific retrocopies.

### Ka/Ks Analysis

For the Ka/Ks analysis, first we extracted coding sequence (CDS) information from the retrocopies and their parental genes based on RefSeq annotation. Next, we executed multiple alignment between the retrocopies and their parental gene sequences using ClustalW2 (Larkin et al. 2007). Finally, the sequence gaps were removed from the multiple alignments, and we used the DNA statistics package (from BioPerl, http://www.bioperl.org/, last accessed June 8, 2015), incorporating the Nei–Gojobori method and the Jukes–Cantor model of nucleotide substitutions, to calculate the Ka and Ks of the multiple alignments.

### Exploring the Genomic Context of Expressed Retrocopies

To understand the genomic context of the retrocopy data sets, we classified the events based on their insertion point: 1) intragenic or intergenic, based on the coordinates of the RefSeq coding and the noncoding transcripts; 2) the polyA proximity (retrocopy insertion <15 kb of a polyA site); and 3) the TSS proximity (retrocopy insertion <15 kb of a known TSS). A permutation test was performed by creating 10,000 random groups of loci with lengths equivalent to the 1,304 expressed retrocopies in humans. Each locus was then classified as either distant or intragenic/near. Finally, we calculated the percentage of intragenic/near events for each random group and compared them to the measured percentage.

### Identification of Expressed Retrocopies

Due to the high similarity between retrocopies and their parental genes, we developed two distinct strategies to reliably detect the set of expressed retrocopies: 1) for intragenic retrocopies, we searched for reads reporting chimeric transcripts that merged host genes and their retrocopies; and 2) for all retrocopies (including intragenic retrocopies) we searched for reads with reliable alignments onto the retrocopies. For both, we used the same RNAseq data set from Brawand et al. (2011).

To detect chimeric transcripts, reads from multiple tissues were aligned to their respective genomes using gsnap (Wu and Nacu 2010) (parameters: -t 30; -B 4; –nofails; -A sam; -m 2; -n 1). Next, we selected reads spanning exonic regions from either, host genes or their intragenic retrocopies. Finally, we selected only those alignments with at least five reads supporting the same chimeric event, alignments defining (putative) introns with canonical splice sites (GT-AG) and an alignment quality higher than 40 (Phred scale). To detect all other expressed retrocopies, we constructed a database containing the sequences and the extra regions from the mature transcripts of the parental genes. This database was created to eliminate false-positive alignments from parental genes. Next, we aligned the reads against this database using bowtie2 (Langmead and Salzberg 2012) (version: 2.0.0-beta6, parameter: –end-to-end; -p 63; -M 40; -D 20; -R 4;-N 0; -L 15; -i S,1,0.50; –ignore-quals), and only reads that aligned uniquely in the retrocopy regions (with an alignment quality >40) were selected and used in the expression analysis.

## Results

### Retrocopies in Primate Genomes

We developed a set of pipelines to identify retrocopies and their parental genes using the reference genome and the known transcriptome for all of the studied species (for further information, see Materials and Methods). Using our computational approach, we identified 57,212 loci originating from the mRNA retrocopies in the seven primates (table 1). For example, we found 7,831 retrocopies in the human genome, of which approximately 91% were also found by the GENCODE consortium or the pseudogene.org database. A similar number of retrocopies (∼7,500, on average) was found in Catarrhini genomes (human, chimpanzee, gorilla, orangutan, and rhesus). In contrast, both Platyrrhini genomes (marmoset and squirrel monkey) presented significantly more retrocopies (∼10,000 events per species), approximately 34% more events than other primates and murine rodents (table 1; *P* value < 2.2e-16, χ^2^ = 449; d.f. = 1).

**Table 1:** Number of Identified mRNA Retrocopies and Their Parental Genes per Species

Species	Number of Retrocopies	Number of Parental Genes
Human	7,831	2,570
Chimpanzee	7,478	2,560
Gorilla	7,706	2,669
Orangutan	6,873	2,439
Rhesus	7,502	2,453
Marmoset	10,465	3,067
Squirrel monkey	9,320	2,864
Mouse	7,109	2,205
Rat	7,364	2,114

**Fig. 1.—:**
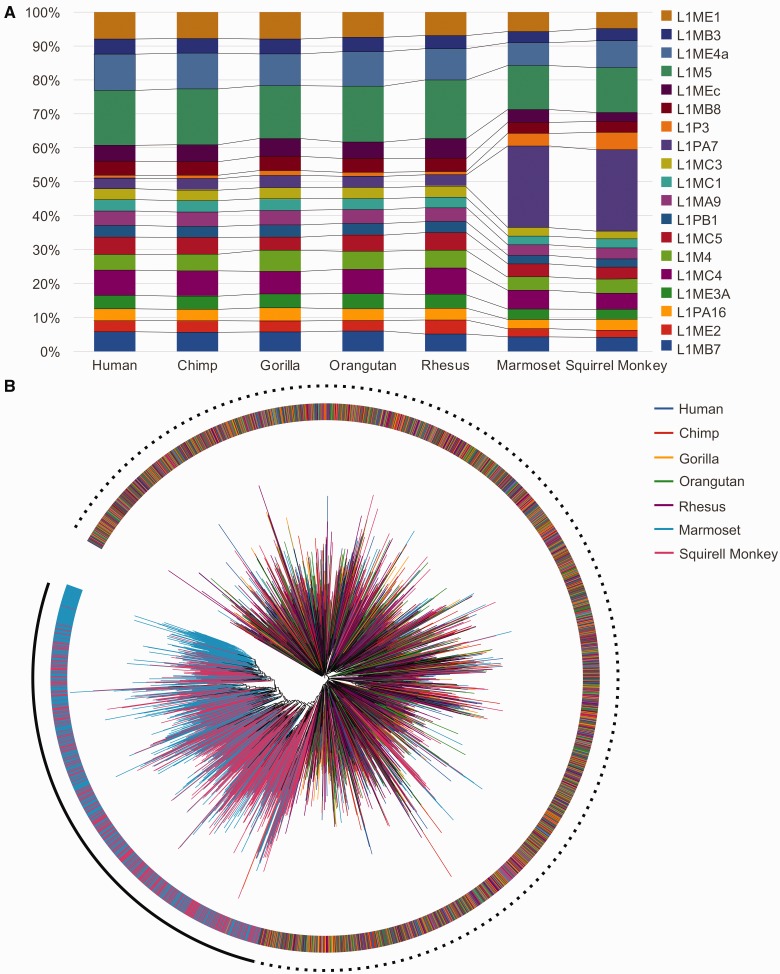
L1 subelement content in the primate genomes. (*A*) The compositions of the most frequent L1 subelements in the primate genomes. (*B*) Phylogenetic tree generated via the multiple alignment of intact L1PA7 ORF2 region. External ring and branch colors are defined by the species from which the sequences were extracted.

To further investigate the larger number of retrocopies in Platyrrhini genomes, we assessed if additional genomic features accounted for the retrocopies enrichment. Compared with Catarrhini, no significant differences were found in the genomic size, the number of genes, the number of transcripts, or the percentage of the genome composed of repetitive elements (supplementary table S1, Supplementary Material online) and the genome assembly qualities (supplementary table S2, Supplementary Material online). Human, chimpanzee, gorilla, orangutan, and rhesus genomes also exhibited a similar composition of L1 subelements, but marmoset and squirrel monkeys presented an overrepresentation of L1PA7 and L1P3 subelements (fig. 1*A*). These two L1 subelements correspond to approximately 25% and 5% of the most frequent L1 elements in NWMs genomes, respectively, but they are significantly less frequent in the Catarrhini genomes (fig. 1*A*), representing only approximately 5% (L1PA7; *P* value < 2.2e-16, χ^2^ = 50,809; d.f. = 1) and approximately 1% (L1PA3; *P* value < 2.2e-16, χ^2^ = 6,913; d.f. = 1), respectively. To elucidate the differences between L1PA7 in Platyrrhini and Catarrhini, we performed a multiple alignment of L1PA7 ORF2p across all complete elements in the seven primate genomes. Despite some similarities in the Platyrrhini and Catarrhini L1PA7 content (suggesting an ancestral origin), the majority of the L1PA7 copies in Platyrrhini do not resemble the L1PA7 in Catarrhini (fig. 1*B*), suggesting a putative lineage-specific expansion of this subelement. Despite the higher content of L1PA7 and L1P3 in the NWMs compared with the OWMs, future studies should be conducted to confirm the contribution of these elements to the larger number of retrocopies in the NWM genomes.

### Retrocopies Shared by Rodent and Primates

In primate genomes, studies based on nucleotide substitutions suggest that most mRNA retrocopies originated within the primate lineage 90–40 Ma, in parallel with a SINE expansion (Ohshima et al. 2003). To further explore this result, we took advantage of the fully sequenced genomes of primates and murine rodents (our outgroup) to precisely identify their shared retrocopies. Due to the identical mechanism of insertion and the large size of primate/rodent genomes, it is reasonable to expect that independent retrotransposition events have distinct genomic insertion points. Consequently, a syntenic genomic locus, sharing the same retrocopied genes, must be the result of an ancestral retrotransposition event. Using this strategy (for details see Materials and Methods and [Navarro and Galante 2013]), we identified 63 (<1%) retrocopies shared among murine rodents and primates that originated before primate-rodent divergence, approximately 90–120 Ma.

By assuming that sequences that are conserved for a long period of time may be functional (Charlesworth et al. 1995), we sought to study these 63 shared primates-rodent retrocopies. First, we found that a majority (50 out of 63 [79%]) of the retrocopies had an annotated RefSeq (Pruitt et al. 2014) transcript (supplementary table S3, Supplementary Material online). Of these 50 retrocopies, 45 were classified as protein-coding genes (i.e., putative retrogenes) for which we identified enrichment for functions related to RNA processing and catabolic processes (supplementary table S4, Supplementary Material online). Additionally, four retrocopies were annotated as noncoding transcripts, and two were annotated as undergoing exonification, that is, forming chimeric transcripts with other genes (supplementary table S3, Supplementary Material online). Moreover, our RNA-Seq analyses (see Materials and Methods and next sections), confirmed that 50 (79%) of the retrocopies are expressed and, as expected for functional retrocopies (Kaessmann et al. 2009), most (96%) are expressed in the testis, including 14 candidates that exhibited tissue-specific expression (supplementary table S5, Supplementary Material online).

Because purifying selection in the genomic sequence can yield powerful evidence of functionality (Lowe et al. 2007), we also evaluated the rate of the nonsynonymous/synonymous (Ka/Ks) distribution of the primate-rodent retrocopies. The 63 retrocopies presented a Ka/Ks distribution with a peak smaller than 0.5 (supplementary fig. S1, Supplementary Material online; median 0.22), whereas 1,000 random sets of 63 retrocopies presented a Ka/Ks centered between 0.5 and 1 (supplementary fig. S1, Supplementary Material online, median 0.58). Such a difference (*P* value < 0.0001; Mann–Whitney *U* test) suggests that most of these retrocopies are subject to selective constraints and, therefore, are potentially functional.

Additionally, we investigated how many of the 63 primate-rodent shared retrocopies are related to the X chromosome, as some genes located on the X chromosome (X-genes) “export” retrocopies to autosomes (Emerson et al. 2004) to escape to X-gene silencing during the haploid stages of spermatogenesis (Richler et al. 1992). In the human genome, we found 43% (27 out 63) of these retrocopies in accordance with this hypothesis, including migrations both out of (expected: 3 retrocopies; found: 13 retrocopies; *P* value = 0.016) and into (expected: 2 retrocopies; found: 14 retrocopies, *P* value = 0.0032) the X chromosome. In comparison, only approximately 1% of all human retrocopies (excluding these 27 retrocopies) were inserted into or originated from genes located in the X chromosome.

### Retrocopy Orthology within Primate Genomes

Based on our results and data from others (Ohshima et al. 2003; Zhang et al. 2004; Marques et al. 2005b), it is clear that most retrocopies in primates originated within their own lineage in the last 90 Myr. However, little is known about retrocopy orthology across primates, and there remains no consensus (Ohshima et al. 2003; Marques et al. 2005b; Pei et al. 2012; Zhang 2013) as to whether they originated over a short period of time during an mRNA retrotransposition burst in an ancestral organism, similar to segmental duplications (Marques-Bonet, Kidd, et al. 2009), or if they were diluted through the primate speciation period (Zhang 2013). To further investigate this question, we attempted to identify ortholog and species-specific mRNA retrotranspositions across the primates.

**Fig. 2.—:**
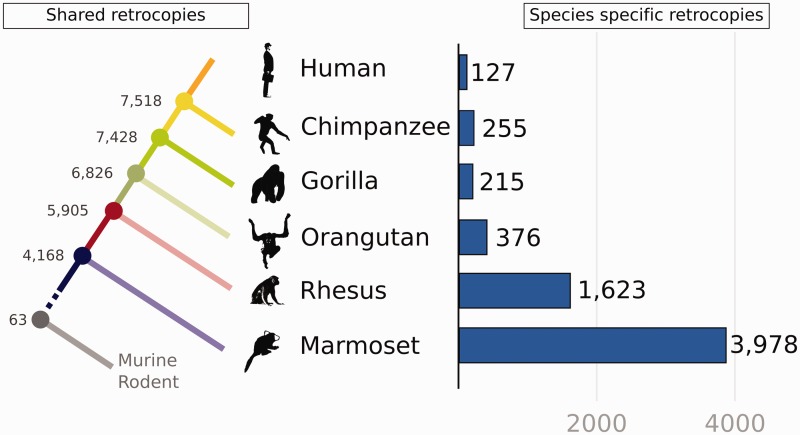
Shared and species-specific retrocopies in primate genomes. The left shows the shared retrocopies. The numbers at the branching nodes represent the retrocopies shared by all of the descendent species that diverged at that point. For example, there are 4,168 retrocopies shared among marmosets, rhesus, orangutans, gorillas, chimpanzees, and humans. The right shows the species-specific retrocopies, which are the retrocopies found only in the respective species.

We identified 4,168 retrocopies that are shared across primates (fig. 2*A*), that is, these retrocopies’ origins date back to before the Platyrrhini-Catarrhini divergence, approximately 42 Ma (Steiper and Young 2006). We also identified 6,134 retrocopies shared between Platyrrhinis, and 7,104 retrocopies shared by humans, chimpanzees, and gorillas (fig. 2*A*). Next, to estimate the rate of retrocopy origination during primate evolution, we estimated the average number of retrocopies that originated within each time period (table 2). We found a continuous decrease in the retrocopy origin and fixation, starting at a higher fixation rate in the primate order (between 42 and 30 Ma), with an average of approximately 142 (1,707/12) retrocopies per million year (table 2 and fig. 2*A*), which decreased only slightly until the great ape lineage (gorilla, chimpanzee and human), corresponding to approximately 68 retrocopies per million year. Curiously, the human lineage shows the smallest rate of species-specific retrocopy origination (table 2 and fig. 2*A*). Otherwise, NWMs have a high rate of retrocopy origination and fixation at approximately 160 retrocopies per million years (table 2 and fig. 2*A*).

**Table 2:** Estimated Rate of Retrocopy Origination/Fixation during Primate Evolution

Evolutionary Period (Ma)	Branch Number	Number of Retrocopies	Divergence Time (Myr)	Average of Retrocopies/Myr
**0–6**	1	127	6	∼21
**6–8**	2	90	2	∼45
**8–18**	3	278	10	∼28
**18–30**	4	731	12	∼61
**30–42**	5	1,707	12	∼142
**0–42**	6	6,734	42	∼160
**42–90**	7	4,105	48	∼85

NOTE.—Branches: 1: the period after the last human/chimpanzee common ancestor; 2: the period after the last gorilla/(chimpanzee, human) common ancestor and before the human/chimpanzee speciation; 3: the period after the last orangutan/(gorilla, chimpanzee, human) common ancestor and before gorilla/(human, chimpanzee) speciation; 4: the period after the last rhesus/(orangutan, gorilla, chimpanzee, human) common ancestor and before orangutan/(gorilla, chimpanzee, human) speciation; 5: in the OWMs lineage, the period after the last NWM/OWM common ancestor and before rhesus/(orangutan, gorilla, chimpanzee, and human) speciation; 6: in the NWMs linage. NWMs retrocopies originated in the period after the last NWM/OWM common ancestor to the present; 7: the period after the last primate/rodent common ancestor and before NWM/OWM speciation.

Next, we investigated the set of species-specific retrocopies. First, we identified candidate retrocopies specific to humans, chimpanzees and gorillas: 127, 255, and 215 retrocopies, respectively (fig. 2*B* and supplementary fig. S2, Supplementary Material online). A selection of the 127 human-specific retrocopies have been described as functional, such as NANOGP8 (Fairbanks et al. 2012), CSNK2A3 (Wirkner et al. 1992), and others (11 events), which remain unfixed in the human population, as we recently described (Schrider et al. 2013). In contrast, larger sets of species-specific retrocopies were found in marmoset (3,978 events) and rhesus (1,623 events) (fig. 2*B* and supplementary fig. S2, Supplementary Material online). Additional details regarding the number of species-specific and shared retrocopies among the primates can be found in the supplementary figure S2, Supplementary Material online. As it is likely that our set of species-specific retrocopies contains false-positive candidates (especially in rhesus and marmoset due to the lack of closely related species), the identification of this set of candidate genes may be an important starting point for further exploration to advance our understanding of species evolution.

### Transcribed Retrocopies in Primates

An increasing number of protein coding and noncoding functional mRNA retrocopies have been reported (Trembley et al. 2005; Tam et al. 2008; Hung et al. 2010; Poliseno et al. 2010; Fairbanks et al. 2012). To be functional, a retrocopy must be transcribed (Kaessmann et al. 2009). To circumvent transcriptional inability, retrocopies generally hijack regulatory elements from other transcribed regions adjacent to their insertion point (Vinckenbosch et al. 2006). Although the ENCODE project has shed light on the stochasticity of the human genome transcriptional capacity, it also suggested that fractions of the expressed retrocopies are not transcriptional noise and may be functional (Pei et al. 2012). Therefore, to extend the set of expressed retrocopies, we used RNA-Seq data (see Materials and Methods) to identify the retrocopies expressed in six healthy tissues (brain, cerebellum, testis, heart, liver, and kidney) from five primates.

**Fig. 3.—:**
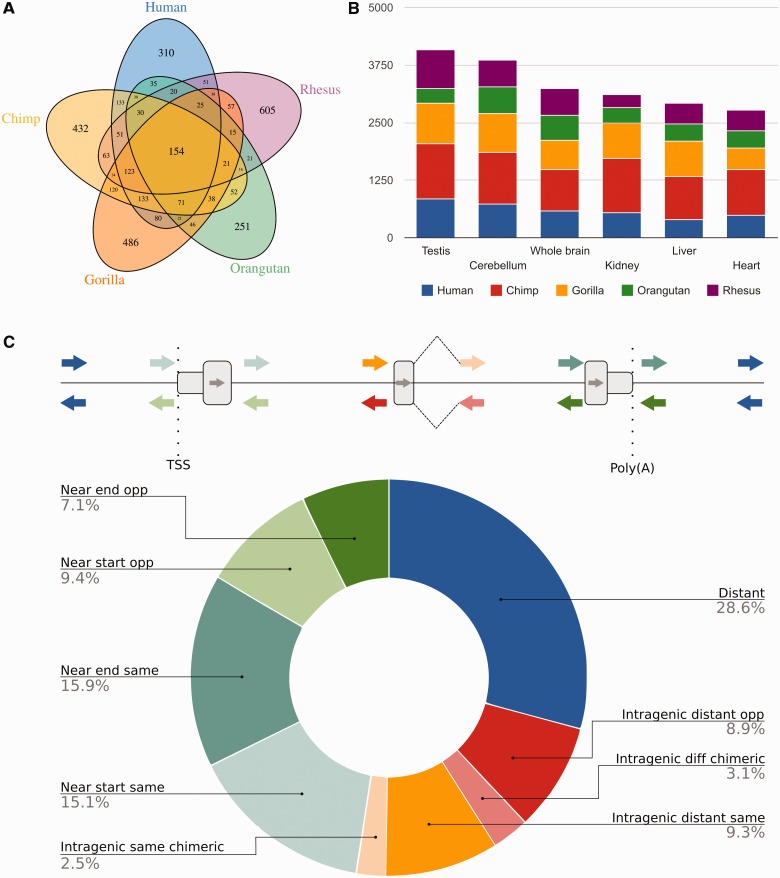
Expressed retrocopies and their genomic context. (*A*) A Venn diagram showing the expressed retrocopies in humans, chimpanzees, gorillas, orangutans, and rhesus. (*B*) Bar plot showing retrocopy expression in various tissues. The retrocopies expressed in two or more tissues were quantified. (*C*) The genomic context for the human expressed retrocopies. The retrocopies were classified according to the chimeric transcript on the same or opposite strand of the host gene (“intragenic same chimeric” and “intragenic different chimeric,” respectively), proximity to the TSS (near TSS), on the same or opposite strand (transcriptional orientation); proximity to the poly(A) site on the same or opposite strand; the intragenic distance from the TSS or the poly(A) site on the same or opposite strand of the host gene, and the distance from the genes.

We identified a large set of expressed 3,562 candidate retrocopies in human (1,304), chimpanzee (1,500), gorilla (1,461), orangutan (846), and rhesus (1,324), figure 3*A*. For most primates, these retrocopies fit the expected gene expression profile already described for humans (Jongeneel et al. 2005). That is, they are more diversified (higher number) in the testis and nervous tissues and less abundant in other highly specialized tissues, such as the kidney, liver, and heart (fig. 3*B*).

To elucidate how these retrocopies were expressed, we analyzed their closeness to regulatory regions. We used human data due to the better genome and transcriptome annotations. As expected (Vinckenbosch et al. 2006), a significant number of these retrocopies (71%; *P* value < 2.2e-16; χ^2^ = 308; d.f. = 2; supplementary fig. S3, Supplementary Material online, Permutation Test, *P* value < 0.0001) were located near or within known genes (fig. 3*C*). Mobilization to another genomic location places the set of expressed retrocopies in a novel transcriptome regulatory context (Kalyana-Sundaram et al. 2012). First, we evaluated the expression profiles of the retrocopies and their parental genes (for human). As expected, we found no correlation between the expression of retrocopies and their parental genes (ρ = −0.0241; *P* = 0.46; Spearman’s correlations; supplementary fig. S4, Supplementary Material online). Next, we evaluated the expression correlation between retrocopies and their hosts or neighboring genes. Interestingly, we found a positive expression correlation for highly expressed retrocopies and their neighboring coding genes for retrocopies located downstream of the coding genes regardless their expected transcriptional orientation, in the same or in the opposite DNA strand (supplementary table S6, Supplementary Material online; ρ = 0.97; *P* value < 2.2e-16; Spearman’s correlations). We also found a positive expression correlation for intragenic retrocopies when both the retrocopies and host genes were transcribed in the same orientation (ρ = 0.92; *P* value = 4.7e-11; Spearman’s correlations), supplementary table S6, Supplementary Material online).

Because genomic loci that exhibit functionalization present species- and/or tissue-specific expression (Vinckenbosch et al. 2006; Bai et al. 2007), we used the index (τ) developed by Yanai et al. (2005) to evaluate the expression breadth of the expressed retrocopies. First, we observed that these retrocopies have an expression profile biased toward tissue-specific when compared with their parental genes (supplementary fig. S5, Supplementary Material online, *P* value < 2.2e-16, Mann–Whitney *U* test). Next, we found 310, 432, 486, 251, and 605 retrocopies exhibiting species-specific expression in humans, chimpanzees, gorillas, orangutans, and rhesus, respectively (fig. 3*A*). Additional analyses are required for in-depth exploration to confirm that our set of transcribed retrocopies contain novel (functional) genes.

## Discussion

Repetitive elements are major actors of genomic plasticity in many species, including primates. A prominent example is LAVA, a novel retrotransposon that emerged exclusively in gibbon (Carbone et al. 2012), and potentially related to the fast karyotype evolution of this ape lineage (Carbone et al. 2014). Several studies have also noted mRNA retrocopies as a source of evolutionary novelty in several species (Ohno 1970; Long et al. 2003; Kaessmann et al. 2009). Here, we performed a systematic analysis of the retrocopies in seven primate genomes (human, chimpanzee, gorilla, orangutan, rhesus, marmoset, and squirrel monkey) and two murine rodents (mouse and rat) and we report their abundance, activity, and expression.

To the best of our knowledge, we provide for the first time an extensive catalogue of their retrocopies found in Old World and New World primates. In agreement with other studies (Baertsch et al. 2008; Balasubramanian et al. 2009; Pei et al. 2012), we found approximately 8,000 retrocopies in the human genome. However, for chimpanzee, orangutan and rhesus, we found twice as many retrocopies as reported by Zhang (2013) in a recent study. This difference emerges from what has already been noted by Baertsch et al. (2008): mRNA-based methodologies (such as we used) are more efficient for identifying retrocopies that are involved in non-CDSs, such as 3′ untranslated regions (3′ UTRs) and noncoding RNAs. However, retrocopy screening based on proteins (used by Zhang) usually revels only half as many candidates. Moreover, due to the high similarity among primate genomes, a similar number of retrocopies between among humans and other primates are expected, such as we identified here.

Platyrrhini is the largest primate family and is composed of approximately 150 species, some of which are endangered on becoming. Most of the Platyrrhini species live in Central and South America (Groves 2001). Furthermore, little is known about these monkeys. For example, their origin in the New World and details of their genome sequences are not fully understood (Jameson et al. 2012). Here, report that marmoset and squirrel monkey (NWMs) have approximately 34% more mRNA retrocopies than OWMs and suggest that this difference may be related to extended L1 subelement activity (L1PA7) into NWMs genomes. In line with our hypothesis, Ohshima et al. (2003) suggested that L1PA7 was one of the top three most probable L1 subfamilies involved in the origination of retrocopies in ancestral primates 40–50 Ma. Despite some indications that more L1PA7 (and L1P3) copies may be related to more mRNA retrocopies in NWMs, we emphasize that additional studies are needed for a complete understanding of the contribution of L1 subelements to the set of mRNA retrocopies in Platyrrhini genomes.

Taking advantage of the access to a rich set of complete primate genome sequences (in addition to nonprimates genomes used as outgroups), we identified retrocopies that are shared by primates and murine rodent (our outgroup) genomes. We showed that more than 90% of primate and murine rodent retrocopies originated independently after the split of their last common ancestors. In agreement with our data, Marques et al. (2005b) and Zhang et al. (2004) previously suggested that most human retrotransposition events occurred after the last human-mouse split, and Ohshima et al. (2003) suggested a burst of retrocopy (and Alus) formations in the genome of ancestral primates approximately 40–50 Ma.

In addition, we also identified 63 retrocopies that are shared between primate and murine rodents. Most of these retrocopies exhibit yield indicators of functionality, such as 1) they were already reported as transcribed genomic regions; 2) they contain an annotated reference mRNA sequence; 3) they appear to be under purifying selection; and d) they are related to the X chromosome, as some migrate out and others into the X chromosome.

Several recent studies have reported an increased number of expressed and potentially functional retrocopies, most of which present not only protein coding (retrogenes) but also noncoding transcripts (Trembley et al. 2005; Baertsch et al. 2008; Tam et al. 2008; Hung et al. 2010; Poliseno et al. 2010; Fairbanks et al. 2012; Kalyana-Sundaram et al. 2012). As expected, a large fraction of these expressed retrocopies are thought to hijack regulatory regions or are inserted into transcribed regions of coding genes (Vinckenbosch et al. 2006). In this study, we used RNA-Seq data and a well-refined gene expression pipeline to expand the set of transcribed retrocopies for primates via the identification of approximately 3,600 transcribed retrocopies in five primates. Some of the retrocopies exhibit tissue specific and noncorrelated expression to their parental genes. We also report a set of intragenic retrocopies that create chimeric transcripts with their host genes, a mechanism for joining protein domains, such as that reported by Vinckenbosch et al (2006). In addition, we identified sets of species- and/or tissue-specific retrocopies, which represents the initial step toward functionalization (Vinckenbosch et al. 2006; Bai et al. 2007). Similarly to Marques et al. (2005b), we identified an enriched set of retrocopies that are expressed in brain and testis tissues, tissues that are essential to the evolutionary success of all animals.

In conclusion, our study has provides at least three major contributions to the retrocopy field. First, we considerably expanded the catalog of mRNA retrocopies for primates, including the identification of large set of retrocopies in Platyrrhini genomes. We also suggested that a portion of the retrocopy content in Platyrrhini is related to extra activity of L1 subelements. Second, we confirmed that most primate and rodent retrocopies originated after their common ancestor. We outlined new details regarding retrocopy origins and conservation across primates and identified a small set of potentially functional retrocopies that are shared by primates and murine rodents. Third, we described a large set of expressed retrocopies, which may contains many coding and noncoding functional retrocopies. In summary, the results presented here may help to unveil how retrocopies can contribute to shaping and creating variability and novelty in the primate genomes.

## Supplementary Material

Supplementary material is available at *Genome Biology and Evolution* online (http://www.gbe.oxfordjournals.org/).

Supplementary Data
