# Supplementary material for: A Genome-Wide Landscape of Retrocopies in Primate Genomes
Source: Genome Biol Evol. 2015 Jul 29;7(8):2265–75. doi: 10.1093/gbe/evv142 (PMC4558860; doi:10.1093/gbe/evv142)
Supplement: Supplementary Data [file supp_7_8_2265__index.html]

A Genome-Wide Landscape of Retrocopies in Primate Genomes — Supplementary Data 

# A Genome-Wide Landscape of Retrocopies in Primate Genomes

## Supplementary Data

files

- Supplementary Data - pdf file
- Supplementary Data - xlsx file
