# Supplementary material for: A Genome-Wide Landscape of Retrocopies in Primate Genomes
Source: Genome Biol Evol. 2015 Jul 29;7(8):2265–75. doi: 10.1093/gbe/evv142 (PMC4558860; doi:10.1093/gbe/evv142)
Supplement: Supplementary Data [file supp_evv142_Supplemental-NavGal.pdf]

Supplemental Material

A genome-wide landscape of retrocopies in primate genomes

Fábio C. P. Navarro and Pedro A. F. Galante

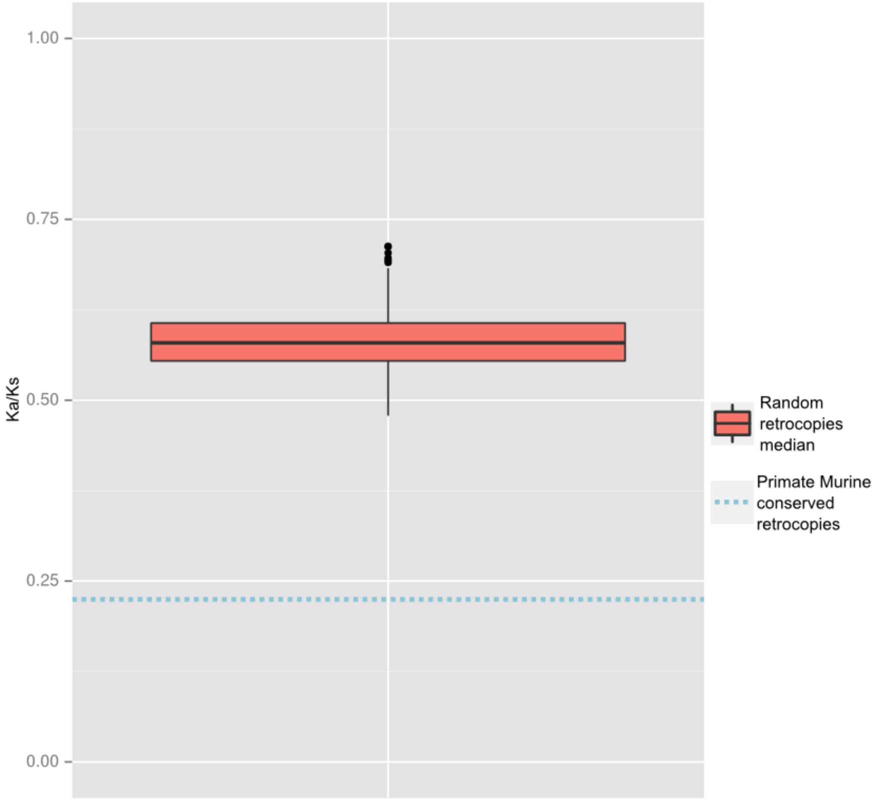

Figure S1. Ka/Ks values of 1,000 random sets of retrocopies and retrocopies shared between rodent (murine) and primates.

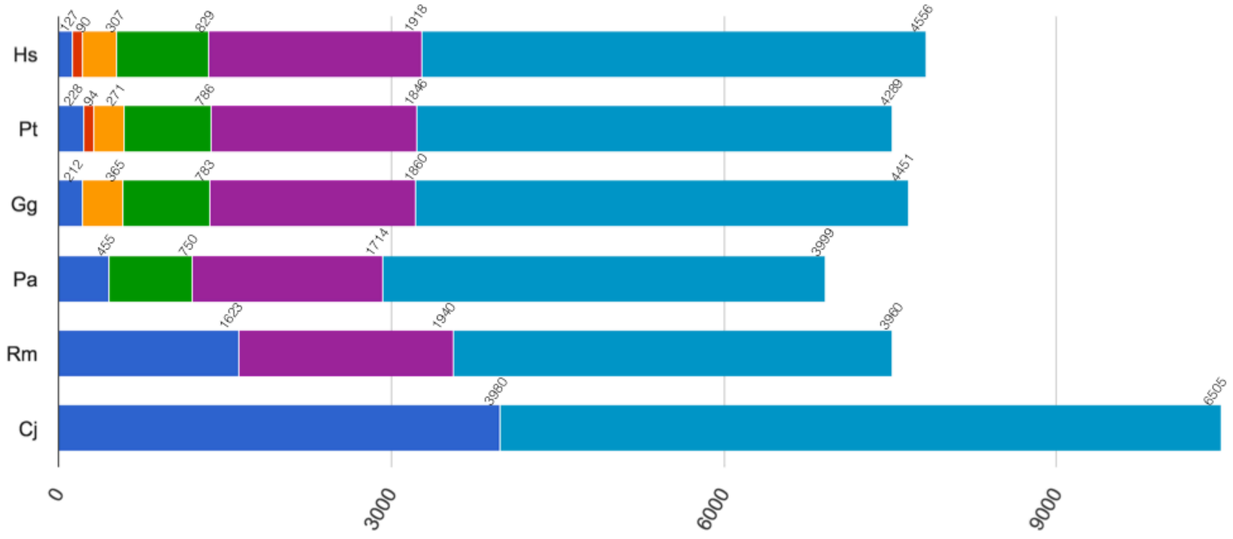

Figure S2. Species-specific and shared retrocopies among primates. Dark blue bars correspond to species-specific retrocopies; Red, yellow, green, purple and light blue bars correspond to retrocopies originated before Human-Chimpanzee Last Common Ancestor (LCA), Human-Chimpanzee-Gorilla LCA, Human-Chimpanzee-Gorilla-Orangutan LCA, Human-Chimpanzee-Gorilla-Orangutan-Rhesus LCA, Human-Chimpanzee-Gorilla-Orangutan-Rhesus-Marmoset LCA, respectively.

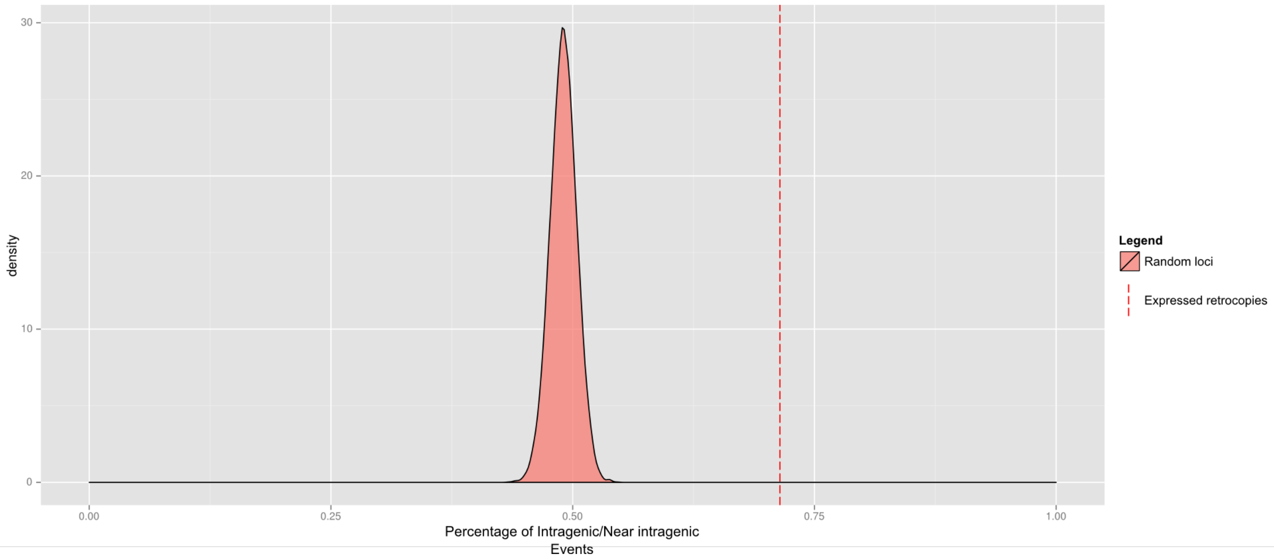

Figure S3. Expressed retrocopies are located near or inside (intragenic) transcribed regions. Percentage of intragenic/near retrocopies from 10,000 randomly selected groups containing 1,304 genomic loci; Dashed line: observed percentage of intragenic/near retrocopies annotated as “expressed retrocopy”.

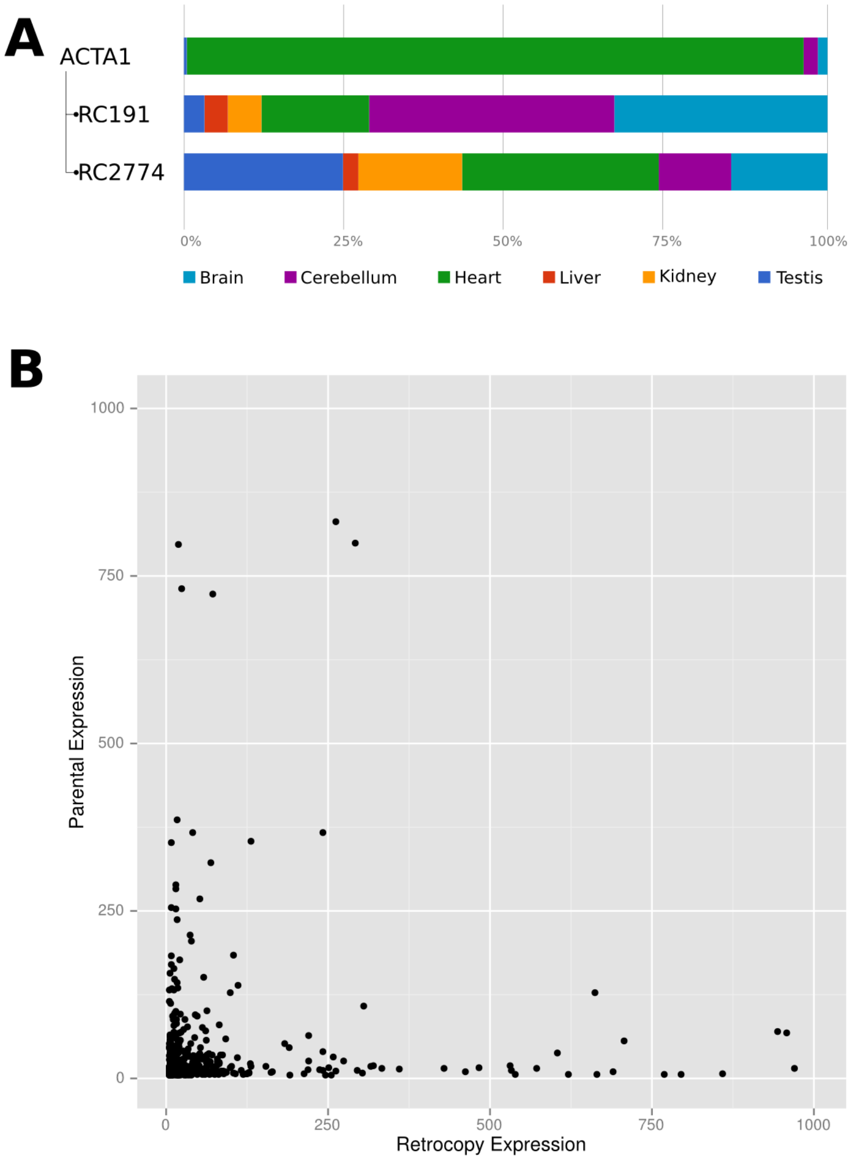

Figure S4. Expression of retrocopies and their parental genes. A) Percent of gene expression in the different tissues for ACTA1 (parental gene) and its two retrocopies (RC191 and RC2774). B) Scatter plot of expression for retrocopies and their parental genes (Spearman correlation = 0.46; p-value = 0.0241).

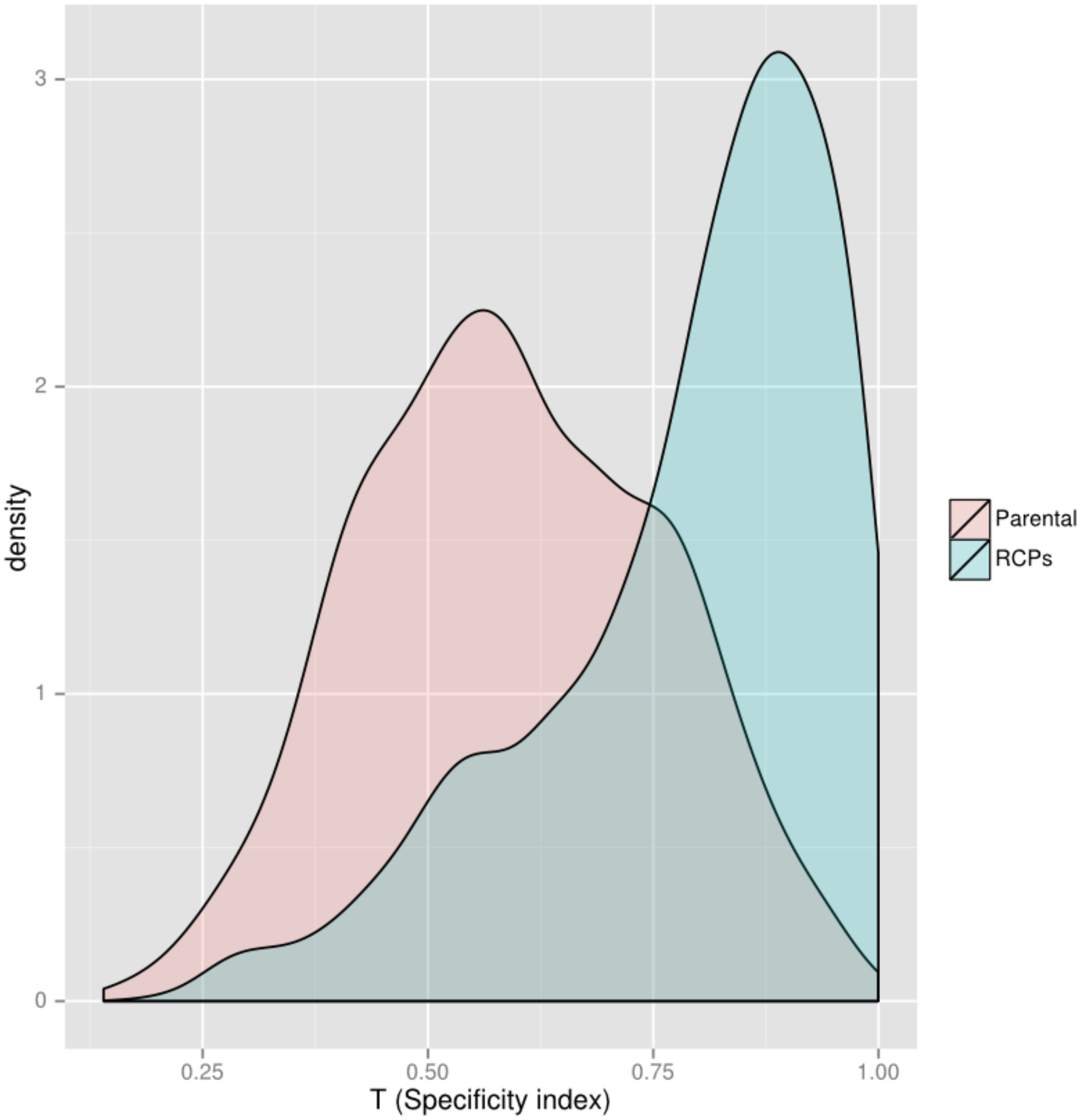

Figure S5. Expression breadth for expressed retrocopies and their parental genes. Here, we used the tissue specificity index (τ) developed by (Yanai et al. 2005), which ranges from 0 (broad expression) to 1 (more tissue specific expression). Retrocopies (RCPs) present more tissue specific expression (p-value < 2.2e-16; Mann-Whitney U test).

Table S1. Primate genomes in summary.

Organism	Genome Size	Number of Genes	Number of Transcripts	Number of Retrocopies	% of genome composed by LINEs/SINEs
**Human**	2.86Gb	19,364	32,201	7,831	22.32% - 13.89%
**Chimpanzee**	2.83Gb	20,998	33,616	7,657	22.23% - 13.66%
**Gorilla**	2.92Gb	20,371	26,821	7,778	20.35% - 11.35%
**Orangutan**	2.94Gb	23,284	28,671	6,962	23.31% - 13.72%
**Rhesus**	2.93Gb	21,018	28,446	7,502	18.86% - 12.54%
**Marmoset**	2.80Gb	18,739	23,275	11,039	21.34% - 13.45%

Table S2. Influence of assembly quality in the detection of retrocopy based on reference genomes.

Assembly name	Organism	Total assembly gap length	Number of scaffolds	Scaffold N50	Contig N50	Number of detected RCPs (including duplications)
**GRCh38**	Human	159970007	735	67794873	56413054	8356
**GRCh37**	Human	239852888	258	46395641	38440852	8335
**NCBI36**	Human	222405369	378	38509590	38509590	8343
**NCBI35**	Human	225449690	379	38509590	38509590	8288
**NCBI34**	Human	226873222	490	29104798	28857747	8263
**NCBI33**	Human	238331975	545	25443670	25443670	8224
**Pan_troglodytes-2.1.4**	Chimpanzee	407207672	27005	8925874	50656	7119
**Pan_troglodytes-2.1.3**	Chimpanzee	407399385	26994	9211238	50595	7657
**Pan_troglodytes-2.1**	Chimpanzee	440199864	32300	8803938	30568	7229

* these data include retrocopies generated by genomic duplications of regions containing retrocopies, which were removed from the retrocopy sets used in the main analyses.

Table S3. Retrocopies shared between primates and rodents. Retrocopy annotation is based on human RefSeq sequences (*i.e.*, all annotated retrocopy has at least a RefSeq sequence).

Parental Gene	Retrocopy annotation	Human	Chimpanzee	Gorilla	Orangutan	Rhesus	Marmoset	Mouse	Rat
FAM133B	FAM133A	X		X	X	X	X	X	X
RPL23A	-	X	X	X		X	X		X
RPL29	-	X	X	X	X	X		X	
ACTG1	-	X	X	X	X	X	X		X
GAPDH	-	X	X	X	X	X	X		X
H3F3A	-	X	X	X	X	X	X		X
HMGB1	-	X	X	X	X	X	X		X
HSPA8	HSPA7 (non-coding)	X	X	X	X	X	X		X
SARNP	CPSF4 (exon)	X	X	X	X	X	X		X
RPS2	-	X	X	X	X	X	X		X
PJA2	PJA1	X	X	X	X	X	X	X	
RPL21	-	X	X	X	X	X	X	X	
OXCT1	OXCT2	X	X	X	X	X	X	X	X
TCEAL6	TCEAL3	X	X	X	X	X	X	X	X
CHM	CHML	X	X	X	X	X	X	X	X
ATXN7L3	ATXN7L3B	X	X	X	X	X	X	X	X
SMEK2	SMEK3P (non-coding)	X	X	X	X	X	X	X	X
CNBP	ZCCHC13	X	X	X	X	X	X	X	X
PCBP2	PCBP1	X	X	X	X	X	X	X	X
PABPC4	PABPC4L	X	X	X	X	X	X	X	X
TMEM151B	TEME151A	X	X	X	X	X	X	X	X
LDHAL6A	-	X	X	X	X	X	X	X	X
TKTL1	TKTL2	X	X	X	X	X	X	X	X
KLHL13	KLHL9	X	X	X	X	X	X	X	X
PDHA1	PDHA2	X	X	X	X	X	X	X	X
GK	GK2	X	X	X	X	X	X	X	X
CSTF2	CSTF2T	X	X	X	X	X	X	X	X
FBL	FBLL1 (non-coding)	X	X	X	X	X	X	X	X
ACTG2	ACTBL2	X	X	X	X	X	X	X	X
ATP6V1E1	ATP6V1E2	X	X	X	X	X	X	X	X
KPNB1	-	X	X	X	X	X	X	X	X
IPO5	RANBP6	X	X	X	X	X	X	X	X
PRPS1	PRPS1L1	X	X	X	X	X	X	X	X
UBA52	UBC	X	X	X	X	X	X	X	X
YY1	YY2	X	X	X	X	X	X	X	X
NAA10	NAA11	X	X	X	X	X	X	X	X

Parental Gene	Retrocopy annotation	Human	Chimpanzee	Gorilla	Orangutan	Rhesus	Marmoset	Mouse	Rat
CRK	-	X	X	X	X	X	X	X	X
FER	THOC2 (exon)	X	X	X	X	X	X	X	X
HNRNPH1	HNRNPF	X	X	X	X	X	X	X	X
HNRNPH1	HNRNPH2	X	X	X	X	X	X	X	X
ACTR3	-	X	X	X	X	X	X	X	X
TUBA3C	TUBAL3	X	X	X	X	X	X	X	X
RPL10	RPL10L	X	X	X	X	X	X	X	X
HSPA8	HSPA1L	X	X	X	X	X	X	X	X
HSPA8	HSPA1B	X	X	X	X	X	X	X	X
EPN1	EPN3	X	X	X	X	X	X	X	X
MKRN1	MKRN3	X	X	X	X	X	X	X	X
SLC25A15	SLC25A2	X	X	X	X	X	X	X	X
USP22	USP27X	X	X	X	X	X	X	X	X
DCAF8	DCAF8L2	X	X	X	X	X	X	X	X
TAF9B	TAF9	X	X	X	X	X	X	X	X
RRAGB	RRAGA	X	X	X	X	X	X	X	X
LPCAT2	-	X	X	X	X	X	X	X	X
MFF	LOC392452 (non-coding)	X	X	X	X	X	X	X	X
NKAP	NKAPL	X	X	X	X	X	X	X	X
DDI2	DDI1	X	X	X	X	X	X	X	X
PAPOLA	PAPOLB	X	X	X	X	X	X	X	X
WDR5	WDR5B	X	X	X	X	X	X	X	X
DNAJB6	DNAJB7	X	X	X	X	X	X	X	X
KCNJ14	KCNJ4	X	X	X	X	X	X	X	X
CHSY3	CHSY1	X	X	X	X	X	X	X	X
MORF4L1	MORF4L2	X	X	X	X	X	X	X	X
GPR153	GPR162	X	X	X	X	X	X	X	X

Table S4. Gene ontology classification (Biological process) for retrocopies shared between rodent and primates.

Category	GO term	Number of retrocopied genes	Percentage of shared retrogenes	Parental gene name
Biological Process	GO:0006397~mRNA processing	6	12.8%	PAPOLB, HNRNPH2, PCBP1, HNRNPF, CPSF4, CSTF2T
Biological Process	GO:0006396~RNA processing	7	14.9%	PAPOLB, HNRNPH2, PCBP1, HNRNPF, FBLL1, CPSF4, CSTF2T
Biological Process	GO:0006986~response to unfolded protein	3	6.4%	HSPA1L, HSPA7, HSPA1B
Biological Process	GO:0051789~response to protein stimulus	3	6.4%	HSPA1L, HSPA7, HSPA1B
Biological Process	GO:0044265~cellular macromolecule catabolic process	6	12.8%	PJA1, USP27X, WDR5B, KLHL9, UBC, HSPA1B
Biological Process	GO:0009057~macromolecule catabolic process	6	12.8%	PJA1, USP27X, WDR5B, KLHL9, UBC, HSPA1B
Biological Process	GO:0019941~modification-dependent protein catabolic process	5	10.6%	PJA1, USP27X, WDR5B, KLHL9, UBC
Biological Process	GO:0043632~modification-dependent macromolecule catabolic process	5	10.6%	PJA1, USP27X, WDR5B, KLHL9, UBC
Biological Process	GO:0000398~nuclear mRNA splicing, via spliceosome	3	6.4%	HNRNPH2, PCBP1, HNRNPF
Biological Process	GO:0000375~RNA splicing, via transesterification reactions	3	6.4%	HNRNPH2, PCBP1, HNRNPF
Biological Process	GO:0000377~RNA splicing, via transesterification reactions with bulged adenosine as nucleophile	3	6.4%	HNRNPH2, PCBP1, HNRNPF
Biological Process	GO:0051603~proteolysis involved in cellular protein catabolic process	5	10.6%	PJA1, USP27X, WDR5B, KLHL9, UBC
Biological Process	GO:0044257~cellular protein catabolic process	5	10.6%	PJA1, USP27X, WDR5B, KLHL9, UBC
Biological Process	GO:0030163~protein catabolic process	5	10.6%	PJA1, USP27X, WDR5B, KLHL9, UBC
Biological Process	GO:0051153~regulation of striated muscle cell differentiation	2	4.3%	MORF4L2, UBC
Biological Process	GO:0051147~regulation of muscle cell differentiation	2	4.3%	MORF4L2, UBC
Biological Process	GO:0006508~proteolysis	6	12.8%	DDI1, PJA1, USP27X, WDR5B, KLHL9, UBC

Table S5. Expression of the rodent/primates shared retrocopies in 5 human tissues (testis, Nervous system (brain and cerebellum), liver, kidney, and heart).

Expression in	Number of human retrocopies
**Testis**	48
**Testis specific**	14
**Brain**	31
**Brain specific**	0
**Cerebellum**	31
**Cerebellum specific**	1
**Liver**	23
**Liver specific**	0
**Kidney**	25
**Kidney specific**	0
**Heart**	29
**Heart specific**	1

Table S6. Expression correlation between expressed retrocopies and their hosts or neighboring genes. Only retrocopies containing more than 40 reads reporting expression were used.

Genomic location	Expected transcription orientation*	Correlation (ρ)	P-value
**Intragenic retrocopy**	Same	0.916834	4.701e-11
**Intragenic retrocopy**	Opposite	-0.2296897	0.2491
**Downstream**	Same	0.9794524	< 2.2e-16
**Downstream**	Opposite	0.9698909	< 2.2e-16
**Upstream**	Same	0.0913412	0.4326
**Upstream**	Opposite	-0.1388423	0.4563

* Expected transcription orientation for retrocopies and hosts or neighboring genes

** Spearman's correlations

Table S7. Number of L1 sub elements in the primate genome.

L1 elements	Human	Chimpanzee	Gorilla	Orangutan	Rhesus	Marmoset
**HAL1**	14884	15033	12219	16136	14482	13339
**L1M4**	10775	11806	13615	14355	11714	11430
**L1M5**	44929	46701	40122	50650	44247	42913
**L1MA9**	10004	10302	9660	11124	9076	8901
**L1MB3**	10811	11020	10451	11866	9565	9600
**L1MB7**	12771	12928	12366	15221	11338	11757
**L1MC4**	16689	17052	14547	18332	16944	15029
**L1MC4a**	16566	16619	6443	18184	15717	13754
**L1MC5**	13456	13615	9995	14385	12725	11974
**L1ME1**	16577	16698	16118	17625	14601	14655
**L1ME4a**	30185	30085	24903	31189	24753	22685
**L1PA7**	10125	10322	9673	11153	9280	83541

Retrocopies annotated by GenCode, pseudogene.org and/or RCPedia (our methods).

To better understand the reliability of our detection of protein-coding retroposition pipeline, we compared the retrocopies identified here (called RCPedia, since these retrocopies are available at RCPedia (Navarro & Galante 2013)) against retrocopies (there, called processed pseudogene) from two databases: (i) pseudogene.org and (ii) GENCODE . Since GENCODE doesn't make available the parental genes, we relied solely on the retrocopy coordinates in the reference genome.

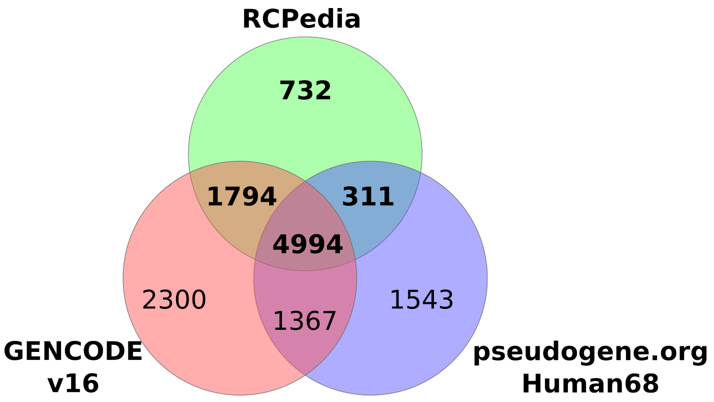

Figure S6. Retrocopies identified and shared by our method (RCPedia), pseudogene.org and GENCODE.

GENCODE and pseudogene.org have 10,455 and 8,215 genomic loci classified as processed pseudogene, respectively. Figure S8 shows that 91% of retrocopies identified by us

have been previously identified. GENCODE and pseudogene.org presented 78% and 81% of its retrocopies also confirmed by other databases. In order to understand better those database specific retrocopies, we performed a comparison between a set of retrocopies from RCPedia and GENCODE. We selected GENCODE because it is the current gold standard of gene and transcript annotation for human currently.

As expected, RCPedia and GENCODE are highly concordant, 6,788 shared retrocopies (87% of RCPedia events), where 3,667 events are specific to GENCODE and potentially false negatives on our database and 1,043 loci are specific to RCPedia, potentially false positives or false negative events in the GENCODE (Figure S8). Given the relative high number of events specific to each method we have manually analyzed 30 and 20 specific events from GENCODE and RCPedia, respectively.

Table S8. Random set of 30 processed pseudogene events specific to GENCODE in comparison to RCPedia.

	Chr	Start	End	Parental Transcript	Length	Manual annotation
1	chr14	19336524	19336668	ENSG00000257721.1	144	Genome duplication
2	chr2	132250386	132277994	ENSG00000152117.13	27608	Unprocessed pseudogene
3	chr19	58175648	58176407	ENSG00000269097.1	759	2 exons – Old retrocopy
4	chr16	31176969	31177248	ENSG00000263343.1	279	2 exons – Old retrocopy
5	chr2	131185304	131186798	ENSG00000230646.1	1494	3 exons – Old retrocopy
6	chr3	20049344	20049739	ENSG00000230697.1	395	Not aligned (using BLAT)
7	chr16	70113032	70113527	ENSG00000241183.1	495	Genome duplication
8	chr9	41776064	41777434	ENSG00000269692.1	1370	Genome duplication
9	chr15	82664459	82748784	ENSG00000237550.4	84325	Genome duplication
10	chr21	15148407	15149587	ENSG00000173231.6	1180	Genome duplication
11	chr22	16122720	16123768	ENSG00000215270.3	1048	Genome duplication
12	chr11	89498052	89498306	ENSG00000255170.2	254	Genome duplication
13	chr12	8559429	8559791	ENSG00000256136.1	362	Genome duplication
14	chr22	36568982	36569996	ENSG00000231576.1	1014	NumTs
15	chr9	42779843	42779998	ENSG00000225433.2	155	NumTs
16	chrX	102061669	102062752	ENSG00000229794.2	1083	NumTs
17	chr12	85333303	85333447	ENSG00000258073.1	144	Repetitive Elements – LTR
18	chr16	34375269	34375779	ENSG00000260449.1	510	Repetitive Elements – Satellite (SST1)
19	chr8	43139769	43139949	ENSG00000253707.1	180	Single exon parental gene
20	chrX	51453887	51454372	ENSG00000223591.4	485	Tandem Duplication
21	chr12	34315397	34315903	ENSG00000256986.1	506	Unknown parental gene
22	chr17	21476800	21477010	ENSG00000265363.1	210	Unknown parental gene
23	chr11	50249920	50250104	ENSG00000255001.1	184	Unknown parental gene
24	chr14	74005925	74006485	ENSG00000258408.1	560	Unknown parental gene
25	chr8	13210910	13211039	ENSG00000253257.1	129	Without exon junction
26	chr4	29909281	29909413	ENSG00000249564.1	132	Without exon junction
27	chr9	41796924	41797395	ENSG00000231511.2	471	Without exon junction
28	chr12	25593809	25593986	ENSG00000255988.1	177	Without exon junction
29	chr2	75825197	75825685	ENSG00000230477.1	488	Without exon junction
30	chrX	27865705	27866056	ENSG00000232834.1	351	Without exon junction

As evidenced by Table S8, most of the unshared events between GENCODE and RCPedia are due to the several reasons. For example, performing a manual alignment using BLAT at UCSC Genome Browser, we found that 6 events (lines #25-#30) classified as processed pseudogene are copies from single exons, which make more difficult to confirm their retrotransposition origin (our method is not able to detect them). We also found four events without known parental genes (#21 to #24), since then the alignment of the annotated loci does not point back to RefSeq sequences or GENCODE transcripts. We also, identified 8 events originated from genomic duplications (#1, #7 to #12) of a retroposition event (which is also discarded by our pipeline). We also identified putative retrocopied loci that gained two or more introns (#2 to #5; also excluded from our candidates). We observed the presence of NumTS (#14 to #16) at the set of processed pseudogene, which we strongly disagree since processed pseudogenes are initially interpreted by literature as loci resulting from a reverse transcription event. Finally, we also found a multi-exonic transcript ranging 27Kb (ENSG00000152117.13) without evidence of being retrocopied and a LTR (#17) annotated as processed pseudogene.

Table S9. Random set retrocopies identified only by RCPedia in comparison to GENCODE. "Details" presents links to their full descriptions at RCPedia web page.

	Chr	Start	End	Parental Transcript	Length	Details
**1**	chr6	35038627	35038826	NM_001016	199	http://www.bioinfo.mochsl.org.br/rcpedia/retrocopies/view/70596
**2**	chr2	8897224	8898480	NM_001177	1256	http://www.bioinfo.mochsl.org.br/rcpedia/retrocopies/view/68126
**3**	chr2	74104255	74105990	NM_022494	1735	http://www.bioinfo.mochsl.org.br/rcpedia/retrocopies/view/67911
**4**	chr6	64190037	64191831	NM_021121	1794	http://www.bioinfo.mochsl.org.br/rcpedia/retrocopies/view/70583
**5**	chr7	44947961	44948360	NM_005274	399	http://www.bioinfo.mochsl.org.br/rcpedia/retrocopies/view/70739
**6**	chrX	56590436	56593256	NM_013438	2820	http://www.bioinfo.mochsl.org.br/rcpedia/retrocopies/view/72013
**7**	chr7	138913182	138913982	NM_001071775	800	http://www.bioinfo.mochsl.org.br/rcpedia/retrocopies/view/71039
**8**	chr1	185301590	185302404	NM_022818	814	http://www.bioinfo.mochsl.org.br/rcpedia/retrocopies/view/65037
**9**	chr22	22457789	22459091	NM_001085411	1302	http://www.bioinfo.mochsl.org.br/rcpedia/retrocopies/view/68683
**10**	chr17	63996465	63997308	NM_005796	843	http://www.bioinfo.mochsl.org.br/rcpedia/retrocopies/view/67216
**11**	chr20	11585629	11589768	NM_024674	4139	http://www.bioinfo.mochsl.org.br/rcpedia/retrocopies/view/68555
**12**	chr9	92324648	92325069	NM_021104	421	http://www.bioinfo.mochsl.org.br/rcpedia/retrocopies/view/71655
**13**	chr11	11202851	11203962	NM_004965	1111	http://www.bioinfo.mochsl.org.br/rcpedia/retrocopies/view/65502

	Chr	Start	End	Parental Transcript	Length	Details
**14**	chr5	94107897	94108107	NM_007209	210	http://www.bioinfo.mochsl.org.br/rcpedia/retrocopies/view/70002
**15**	chr2	65860969	65861129	NM_015933	160	http://www.bioinfo.mochsl.org.br/rcpedia/retrocopies/view/67912
**16**	chr2	70315029	70316278	NM_001128912	1249	http://www.bioinfo.mochsl.org.br/rcpedia/retrocopies/view/67853
**17**	chr17	63996465	63997308	NM_005796	843	http://www.bioinfo.mochsl.org.br/rcpedia/retrocopies/view/67216
**18**	chr11	56098383	56100015	NM_016255	1632	http://www.bioinfo.mochsl.org.br/rcpedia/retrocopies/view/65718
**19**	chr8	74743365	74743721	NM_002925	356	http://www.bioinfo.mochsl.org.br/rcpedia/retrocopies/view/71222
**20**	chr12	25070653	25071266	NM_001344	613	http://www.bioinfo.mochsl.org.br/rcpedia/retrocopies/view/66217

We manually analyzed 20 random retrocopies identified only by RCPedia (Table S9). Initially, we observed that, at least, two events annotated by our pipeline are described as protein-coding genes in GENCODE. As we stated, we do not make the distinction of pseudogenes and genes since we are interested on the general aspects of retroduplications and not at solely pseudogene specific features. We also found 4 (4,9,18,19) events annotated as unprocessed pseudogene and ambiguous ORF on loci that are clearly decedent of reverse transcription reaction, since they are intronless and present parental multi-exonic genes. The remaining events were all missed by GENCODE pipeline. While most of the events we could not explain the absence in the GENCODE, we see at least 3 events containing a high proportions of repetitive sequences. Therefore, despite the evident absence of false positives retrocopies on this set of 20 randomly selected retrocopies, we do not believe that all of our 1,043 specific events (not found in GENCODE) are true positive. We expect a small number (0.05%) of false positive candidates (Table S9).

Taken together these results highlight the complexity of annotating retrocopied loci. Even the two main standards for processed pseudogene (GENCODE and pseudogene.org) are far from being concordant. Here and in our previous publication (Navarro & Galante 2013), we try to shed light on this complexity and contribute to a robust base for future works in the retrocopy area.

References

Navarro FCP, Galante PAF. 2013. RCPedia: a database of retrocopied genes. Bioinformatics. 29:1235–1237. doi: 10.1093/bioinformatics/btt104.

Yanai I et al. 2005. Genome-wide midrange transcription profiles reveal expression level relationships in human tissue specification. Bioinformatics. 21:650–659. doi: 10.1093/bioinformatics/bti042.
